# Harnessing open science practices to teach ecology and evolutionary biology using interactive tutorials

**DOI:** 10.1002/ece3.11179

**Published:** 2024-05-30

**Authors:** Jory E. Griffith, Elizabeth Houghton, Margaret A. Slein, Maxime Fraser Franco, Jhoan Chávez, Amy B. Forsythe, Victoria M. Glynn, Egor Katkov, Kirsten M. Palmier, Zihaohan Sang, Rolando Trejo‐Pérez, Bryn Wiley, Jennifer M. Sunday, Joey R. Bernhardt

**Affiliations:** ^1^ Department of Biology McGill University Montreal Quebec Canada; ^2^ Department of Biology University of British Columbia (Okanagan) Kelowna British Columbia Canada; ^3^ Department of Zoology University of British Columbia Vancouver British Columbia Canada; ^4^ Département des Sciences Biologiques Université du Québec à Montréal Montreal Quebec Canada; ^5^ Department of Geography, Earth, and Environmental Sciences University of Northern British Columbia Prince George British Columbia Canada; ^6^ Redpath Museum and Department of Biology McGill University Montreal Quebec Canada; ^7^ Smithsonian Tropical Research Institute Balboa Republic of Panama; ^8^ Department of Biology University of Regina Regina Saskatchewan Canada; ^9^ Department of Computer Science University of Toronto (St. George) Toronto Ontario Canada; ^10^ Institut de Recherche en Biologie Végétale Université de Montréal Montreal Quebec Canada; ^11^ Department of Integrative Biology University of Guelph Guelph Ontario Canada

**Keywords:** cognitive load theory, educational resources, online tutorials, open science, R programming, student‐centered learning, teaching, undergraduate education

## Abstract

Open science skills are increasingly important for a career in ecology and evolutionary biology (EEB) as efforts to make data and analyses publicly available continue to become more commonplace. While learning core concepts in EEB, students are also expected to gain skills in conducting open science to prepare for future careers. Core open science skills like programming, data sharing, and practices that promote reproducibility can be taught to undergraduate students alongside core concepts in EEB. Yet, these skills are not always taught in biology undergraduate programs, and a major challenge in developing open science skills and learning EEB concepts simultaneously is the high cognitive load associated with learning multiple disparate concepts at the same time. One solution is to provide students with easily digestible, scaffolded, pre‐formatted code in the form of vignettes and interactive tutorials. Here, we present six open source teaching tutorials for undergraduate students in EEB. These tutorials teach fundamental ecological concepts, data literacy, programming (using *R* software), and analysis skills using publicly available datasets while introducing students to open science concepts and tools. Spanning a variety of EEB topics and skill levels, these tutorials serve as examples and resources for educators to integrate open science tools, programming, and data literacy into teaching EEB at the undergraduate level.

## Graphical abstract

Open science skills are becoming increasingly important in academic and industry working environments in the fields of ecology and evolutionary biology (EEB). Yet, learning EEB concepts alongside open science skills such as programming is challenging for undergraduate students. To address this issue, we developed six open‐access interactive tutorials integrating open science tools, programming, and data literacy into undergraduate teaching on topics of ecology and evolution.

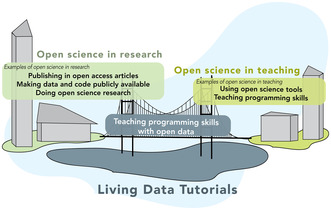

## 1 INTRODUCTION

The open science movement represents a paradigm shift in the way we do science, emphasizing the sharing of data, analyses, and ideas to promote reliability, reproducibility, transparency, and collaboration during the scientific process (Rinaldi, 2014). However, practicing open science often requires an additional set of skills, including familiarity with open‐source programming languages (e.g., *R, Python, Julia*), reproducible workflows, version control systems (e.g., Git), and data/code sharing platforms (e.g., Dryad, GitHub, Open Science Framework, Figshare, etc.) (Hampton et al., 2015). As these skills become increasingly important for participation in research and the workforce, universities have begun to incorporate them into their curricula (e.g., Hanna et al., 2021; Jekel et al., 2020; Pownall et al., 2023; Read et al., 2023). Initiatives that have developed materials to incorporate open science skills into science curricula highlight open science practices as an essential part of the scientific process (e.g., CIEE‐ICEE, 2023; Openscapes, 2023; Schmidt et al., 2016; Teal et al., 2015).

Teaching students fundamental ecology and evolutionary biology (EEB) concepts alongside technical open science skills can be challenging (Guzman et al., 2019). Effectively balancing multiple skills to ensure that students can incorporate multifaceted information easily is an issue addressed by cognitive load theory (Sweller et al., 2019, 1998). Cognitive load theory recognizes that learners can only process a limited amount of information at a time, and works to mitigate the effort required to understand multiple concepts and commit them to long‐term memory (Ou et al., 2022; Sweller et al., 2019). For example, the combination of teaching programming skills and biological concepts simultaneously can decrease student engagement with the course content due to high cognitive load (Engels et al., 2023; Guzman et al., 2019). Additionally, teaching introductory open science practices alongside discipline‐specific scientific content in undergraduate courses can be challenging for instructors due to the need to teach programming skills and theoretical concepts simultaneously. Instructors may need to account for varied programming skill levels and learning speeds, which requires a flexible teaching approach (Farrell & Carey, 2018). Another challenge instructors may face is that they may lack the necessary knowledge and training to teach data science skills to undergraduate students (Emery et al., 2021). The fields of EEB have changed drastically in a short period of time due to technological advances that facilitate the handling of large amounts of data, such as an increase in the use of programming languages and version control systems (Hampton et al., 2013; Michener & Jones, 2012). Thus, many instructors may not have the time to keep up with rapidly evolving methods and develop up‐to‐date data science curricula for their undergraduate courses.

One strategy to overcome these challenges for students and instructors is the use of publicly available, self‐paced, and scaffolded tutorials. In scaffolded tutorials, structure is provided to assist with introducing new concepts and skills that students can then apply at their own pace (Van De Pol et al., 2010). Using scaffolded interactive tutorials that cater to a variety of student proficiency levels (e.g., programming skills or statistical background) can reduce cognitive load and improve student engagement and comprehension when learning programming and discipline‐specific content. This approach allows students to engage with material most comfortable to them, whether that means directly writing associated code or adjusting data inputs in an interactive application (Custer et al., 2021; Farrell & Carey, 2018; McGuire et al., 2022; Rissanen & Costello, 2023). A self‐paced, scaffolded, multi‐proficiency level approach to learning empowers students to challenge themselves in a productive way by tackling complex problems while gaining computational literacy and scientific skills (Dill‐McFarland et al., 2021; Emery et al., 2021; Farrell & Carey, 2018). Moreover, publicly available, well‐documented teaching tutorials with explicit guidelines can help instructors develop course content without the need for complex technical skills, while also providing a template for instructors to develop their own course content (Emery et al., 2021; Farrell & Carey, 2018). Therefore, using scaffolded tutorials to teach programming alongside discipline‐specific content in the classroom can be an effective strategy to optimize teaching and learning multiple concepts simultaneously.

Integrating publicly available datasets into scaffolded tutorials can introduce students to answering scientific questions using real‐world data (Greengrove et al., 2020; O'Reilly et al., 2017; Styers et al., 2021). The ability to work with large open‐source datasets is becoming increasingly important as the use of these datasets in ecological research continues to rise (Culina et al., 2020; Michener & Jones, 2012; Roche et al., 2022). Previous work has integrated publicly available data to teach undergraduate and graduate students foundational concepts in their respective fields (e.g., oceanography via oceanographic time series [Greengrove et al., 2020], GIS/remote sensing via satellite data [Styers et al., 2021], earth and environmental science via lacustrine records [O'Reilly et al., 2017]). Students reported feeling more comfortable with ecological concepts and the analysis of unstructured data after completing self‐directed tutorials featuring real‐world ecological data (O'Reilly et al., 2017; Styers et al., 2021). In addition, students reported feeling a larger appreciation for publicly available datasets and an increased awareness of their potential uses, which could promote future engagement with open data practices (O'Reilly et al., 2017). Together, these studies demonstrate the benefits of integrating publicly available data with EEB concepts in undergraduate courses for skill building, knowledge retention, and the use of open science practices (LaDeau et al., 2017).

Here, we present a series of *R*‐based (R Core Team, 2022) tutorials that integrate programming skills with EEB concepts using publicly available data from Canada. We developed six free and openly accessible teaching tutorials with the following goals: (1) Provide instructors with open source teaching tutorials, guidelines for implementation, and a framework that can be modified to apply to their own courses, (2) Teach EEB concepts while mitigating cognitive load by allowing for students to primarily focus on exploring biological concepts, with the option to engage with statistical and/or programming skills secondarily, and (3) Use publicly available datasets to teach students how to explore and analyze real‐world data. We summarize the tutorials below, expanding on two selected tutorials, and suggest how they can be integrated into undergraduate classrooms.

## 2 BACKGROUND: OPEN ACCESS TUTORIALS

**FIGURE 1:**
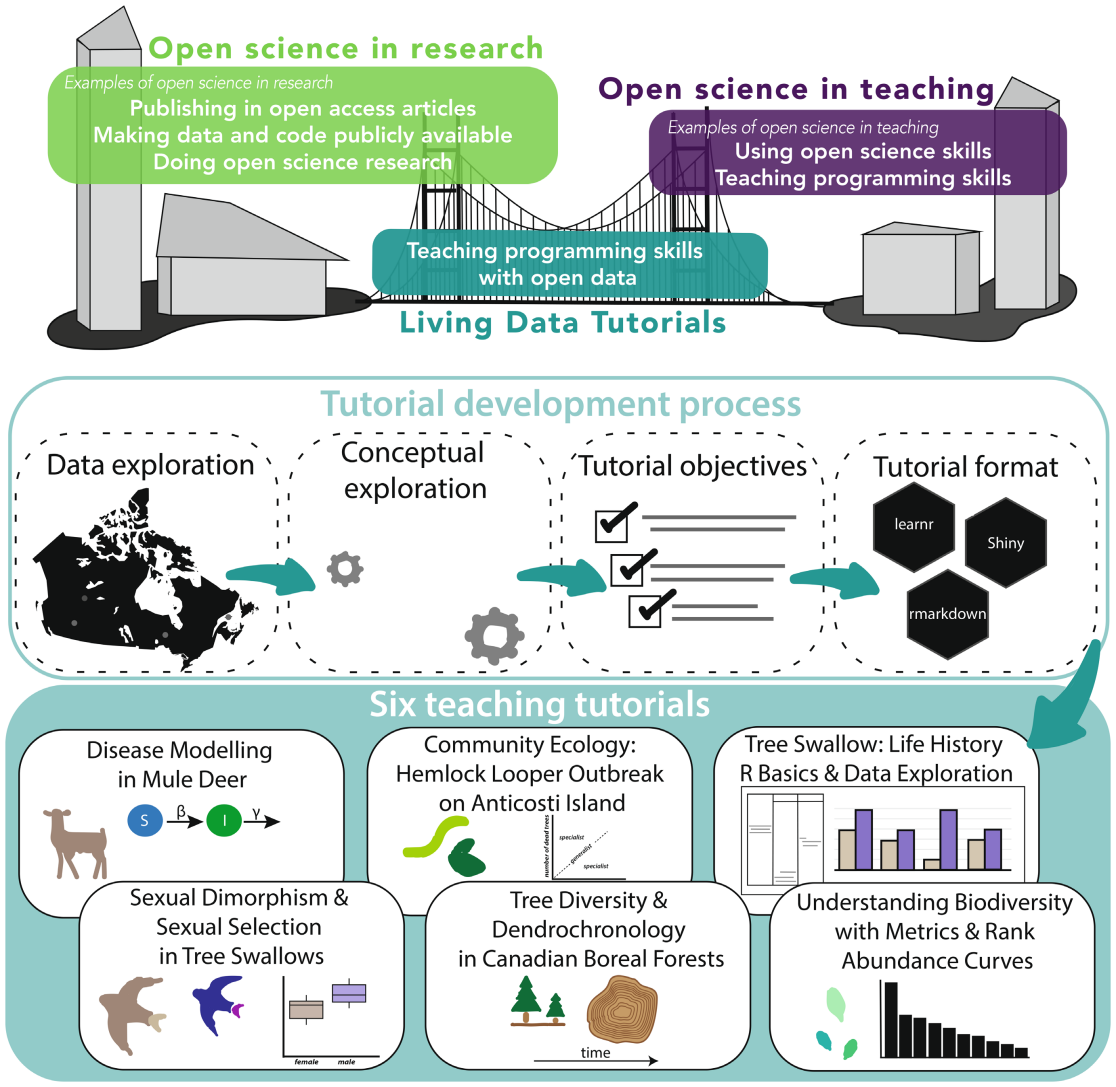
Schematic of the goals, processes, and summary of the teaching tutorials created as part of the Living Data Tutorials. The upper panel shows the overarching goal to link open science in research and open science in teaching, the middle panel shows the process of tutorial development (data exploration, conceptual exploration, tutorial objectives, and tutorial format), and the lower panel shows the six undergraduate teaching tutorials.

A group of twelve graduate students, three post‐doctoral facilitators, and six faculty members from universities across Canada participated in an online working group titled *Data Bytes in Ecology and Evolutionary Biology* hosted by the Living Data Project (LDP). The LDP is a nationwide initiative organized by the Canadian Institute for Ecology and Evolution/Institut Canadien d'Écologie et d'Évolution (CIEE/ICEE) to teach and promote open science practices for graduate students through data rescue (i.e., identifying, preserving, and sharing valuable data and metadata at risk of loss; Bledsoe et al., 2022), education, and working groups. As a Canadian initiative, this working group was formed with the goal of developing openly accessible pedagogical tutorials for undergraduate students on foundational concepts in EEB using open source or rescued datasets collected in Canada (Figure 1).

We developed six tutorials using *R* with five datasets from Canadian ecosystems. Tutorials covered various EEB concepts (e.g., population dynamics, disease ecology, biodiversity indices, ecological specialization) and were written for students with diverse proficiency levels (Table 1). We provided recommendations for undergraduate course levels (from first year to fourth year) that these tutorials would best support based on the type of content featured in EEB course descriptions at Canadian universities (Table 1). To address multiple programming skill levels, tutorials also featured various coding formats and complexity (e.g., Shiny [Chang et al., 2021], R Markdown [Allaire et al., 2023], and learnR [Aden‐Buie et al., 2023]) (Table 1). All tutorials can be accessed online at https://living‐data‐tutorials.github.io.

**TABLE 1:** Overview of the Living Data Tutorials, a set of open access teaching tutorials for use in undergraduate EEB curricula (https://living‐data‐tutorials.github.io).

Tutorial	Source dataset	Learning objectives	Ecological Topics	Format	Suggested audience	Suggested courses	Estimated time for completion
(A) Tree Swallow Life History: R Basics & Data Exploration	Tree Swallow nest box productivity data set from Long‐Point, Ontario, Canada (1977–2014) (Diamond et al., 2021)^a^	Explore and apply basic commands for handling ecological data in *R* Practice plotting data Observe trends and conclusions from figures	Data exploration	R Markdown	First year	Introduction to Evolution and Ecology Organisms and the Environment	30–45 min
(B) Community Ecology: Hemlock Looper Outbreak on Anticosti Island	Secondary insects and fungi post‐hemlock looper on Anticosti in 1973 (Government of Canada, 2021)^a^	Interpret community ecology plots Differentiate between a generalist and specialist species using community ecology plots Make data‐driven management recommendations for hemlock looper	Species dynamics, tree communities, host preference, specialist vs. generalist	learnR Shiny	First & second year	Ecology and Evolution Organisms and the Environment	45–60 min
(C) Sexual Dimorphism & Sexual Selection in Tree Swallows	Tree Swallow nest box productivity data set from Long‐Point, Ontario, Canada (1977–2014) (Diamond et al., 2021)^a^	Identify sexual dimorphism Explore how life history traits drive evolutionary trends Evaluate population dynamics in tree swallows	Sexual dimorphism, life history, trade‐offs	R Markdown	Second year	Ecology and Evolution	30–45 min
(D) Tree Diversity & Dendrochronology in Canadian Boreal Forests	Seasonal and annual dynamics of western Canadian boreal forest plant communities (Hesketh et al., 2021)^a^	Estimate and visualize plant diversity variation across space and environment Explore linear and non‐linear model relationships using ecological data Estimate and visualize tree ring dynamics over time	Biodiversity, tree morphology and growth	learnR Shiny	Second & third year	Ecological Dynamics	45–60 min
(E) Disease Modelling in Mule Deer	Chronic wasting disease in deer (history in Alberta) (Government of Alberta, 2023)	Explore susceptible‐infected (SI) models and their application in predicting disease spread Investigate the role of R_0_ in determining disease spread Explore how vaccination and knowledge of *R*_0_ can be used to prevent the spread of disease	Disease ecology, population dynamics	Shiny	Third year	Population Biology Ecology Mathematical Biology	30–45 min
(F) Understanding Biodiversity with Metrics & Rank Abundance Curves	Turkey lakes watershed study (Government of Canada, 2020)^a^	Identify, calculate, and implement commonly used metrics to quantify biodiversity Analyze biodiversity change in benthic invertebrates over time and after disturbance	Rank‐abundance curves, species richness, diversity indices	R Markdown	Third & fourth year	Ecology and Evolution Community or Aquatic Ecology	45–60 min

*Note*: Each tutorial was developed using a publicly available Canadian dataset to teach ecology and evolution concepts with specific learning objectives. Each tutorial has a suggested level(s) and undergraduate course(s).

^a^ Datasets rescued through the Living Data Project (LDP) data rescue internship program.

One goal of the tutorials was to mitigate cognitive load when teaching EEB concepts alongside *R* programming. For example, some tutorials offer the option to hide or show *R* code (e.g., the “Community Ecology: Hemlock Looper Outbreak on Anticosti Island” tutorial and provide students with options to practice coding independently (e.g., the “Tree Swallow Life History: R Basics & Data Exploration” tutorial). Other tutorials use a Shiny application (Shiny App) to eliminate engagement with the code entirely to reduce cognitive load while still allowing students to engage with dynamic data visualization, modeling, and interpretation of results (e.g., the “Disease Modelling in Mule Deer” tutorial). These tutorials also introduce a range of *R* tools and supporting packages (e.g., “codyn” [Hallett et al., 2020], “tidyverse” [Wickham et al., 2019], etc.). To assist instructors in integrating these tutorials into course curricula, we have included sample guiding questions and lesson plans as an accompaniment to the tutorials on the tutorial website (see “Guidelines for instructors” tab on the website https://living‐data‐tutorials.github.io/).

## 3 EXAMPLE APPLICATIONS FROM SELECT TUTORIALS

**FIGURE 2:**
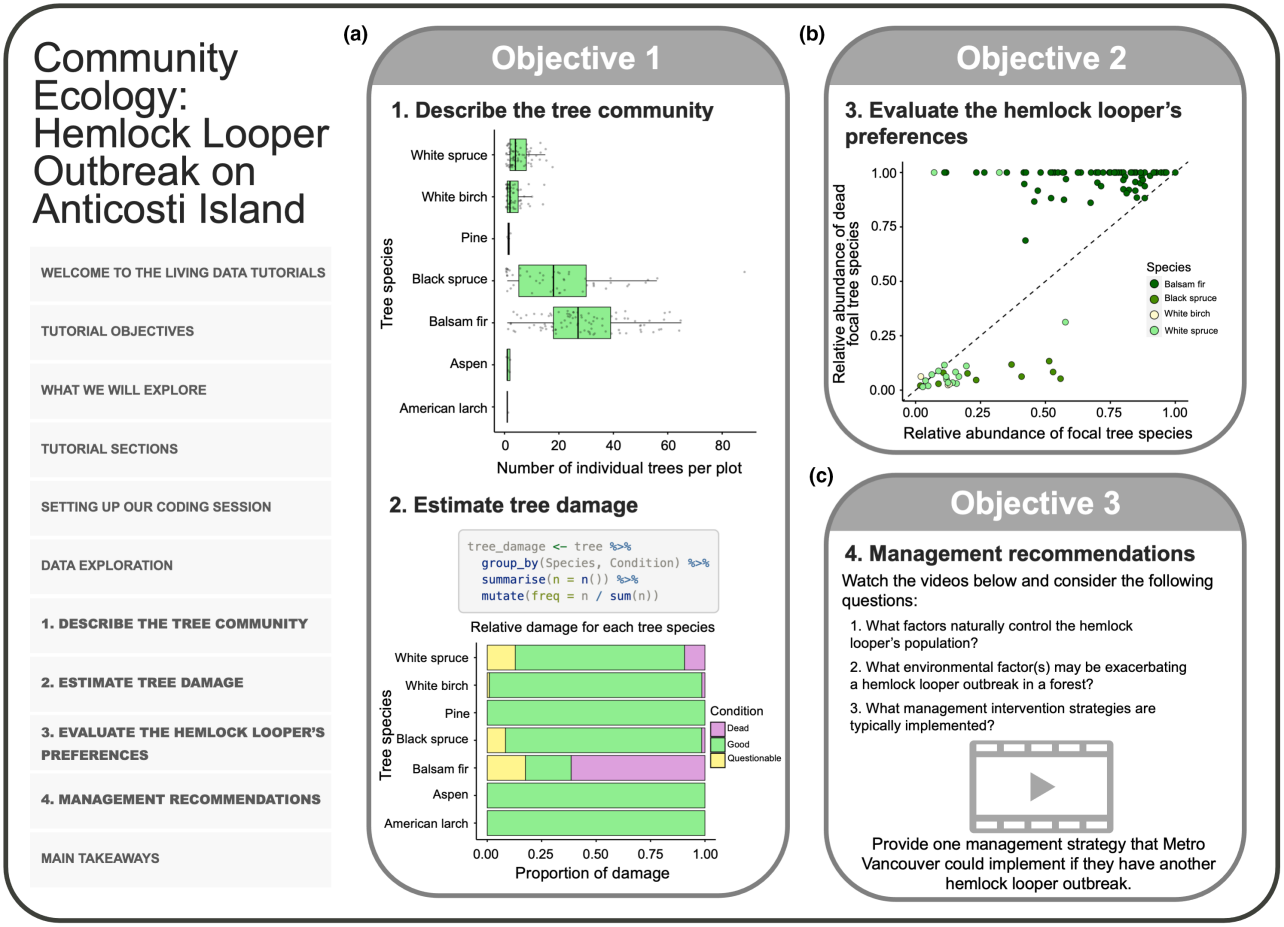
Summary of content, examples, and knowledge testing questions covered in the tutorial, “Community Ecology: Hemlock Looper Outbreak on Anticosti Island”. (a) Students interpret community ecology plots by describing tree communities using box plots of species' abundances and using bar graphs to estimate the proportion of each tree species that was damaged by the hemlock looper (Objective 1). (b) Students evaluate the hemlock looper's tree species preferences by plotting tree species' mortality against tree species' abundance (Objective 2). (c) Students make management recommendations in other geographical regions based on information covered throughout the tutorial and suggested videos provided in this section (Objective 3).

**FIGURE 3:**
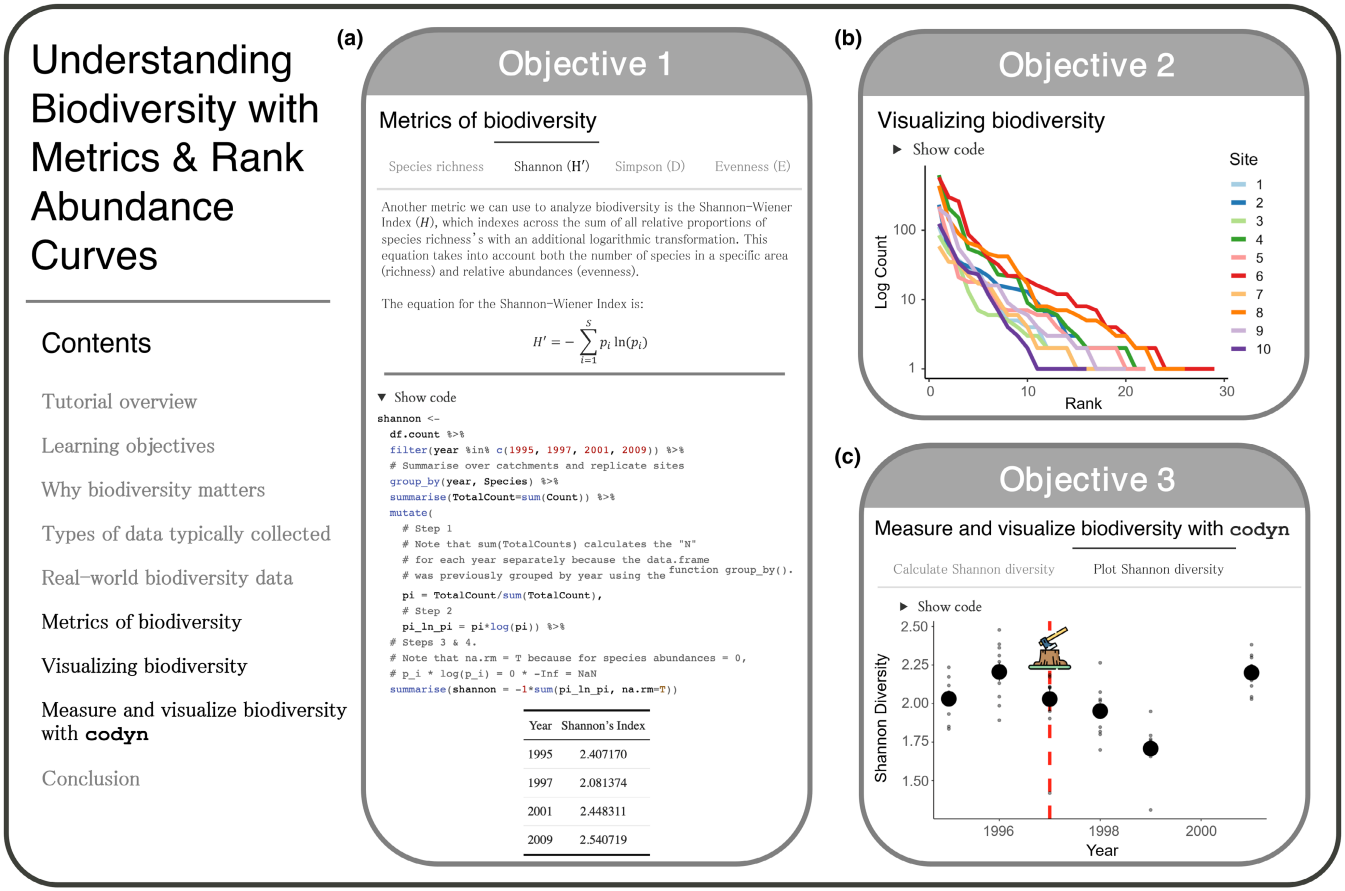
“Understanding Biodiversity with Metrics & Rank Abundance Curves” tutorial content and examples of the material and exercises. Students are taught to (a) identify, calculate, and implement commonly used metrics (richness, Shannon and Simpson diversity, and evenness) to quantify biodiversity (Objective 1), (b) understand and create rank abundance curves to visualize biodiversity (Objective 2), and (c) use the *R* package “codyn” to calculate diversity metrics and plot over time to visualize biodiversity change following disturbance (Objective 3).

Here, we highlight two of these six tutorials to show how we teach EEB concepts using open data and real‐world examples. We demonstrate how these tutorials mitigate cognitive load, accommodate multiple proficiency levels, and can be applied in the classroom (Table 1). We introduce two tutorials: (1) “Community Ecology: Hemlock Looper Outbreak on Anticosti Island” and (2) “Understanding Biodiversity with Metrics & Rank Abundance Curves” to demonstrate the variety of layouts in the tutorials (see Figures 2 and 3). In each tutorial example, we include a tutorial overview, background of data collection for the datasets featured, description of the tutorial activities, expansion on three tutorial learning objectives, and potential tutorial applications.

### 3.1 Tutorial example 1. Community ecology: Hemlock looper outbreak on Anticosti Island

#### 3.1.1 Tutorial overview

In this tutorial, we present a broad introduction to consumer‐resource interactions using a case study of the hemlock looper (*Lambdina fiscellaria*), a lepidopteran that feeds on trees during its larval stages (Table 1, row B). We used a publicly available dataset reporting tree damage during a hemlock looper outbreak on Anticosti Island, Québec, Canada in 1973 (Government of Canada, 2021). This tutorial aims to teach students how to extract ecological information from a dataset (e.g., species abundances and food preferences) and make inferences given the information, using primary data science exploration and visualization techniques (Figure 2a; see the tutorial section one: “Describe the tree community” and two: “Estimate tree damage”). In addition, the tutorial asks students to use their gained insights into the hemlock looper's ecology (Figure 2b; see tutorial section three: “Evaluate the hemlock looper's preference”) to make data‐driven management recommendations for an outbreak in a new location: Metro Vancouver, British Columbia (Figure 2c; see tutorial section four: “Management recommendations”). Together, this tutorial presents the ecological and socioeconomic ramifications of interactions between insect pests and their tree hosts.

#### 3.1.2 Data collection background

During the outbreak on Anticosti Island, researchers visually surveyed the degree of damage caused by the hemlock looper. The species of each tree affected by the hemlock looper was recorded, and each tree was classified by herbivory status. Trees were classified as “Good” if they did not show any signs of damage from the outbreak, “Dead” if the hemlock looper had attacked and killed the tree, or “Questionable” if the surveyors were unsure. The data are available on the Government of Canada's open data portal (Government of Canada, 2021).

#### 3.1.3 Description of the activity

Using the hemlock looper consumer‐resource case study as a framework, the tutorial covers basic ecological concepts such as species' absolute and relative abundances, tree community composition after a pest outbreak, and ecological specialization (i.e., generalist and specialist species). These concepts are covered across four sections in the tutorial that address three distinct learning objectives (featured below). Throughout the tutorial, students can view well‐annotated *R* code used to explore the data, create plots, and calculate summary statistics. At the end of each section, students can test their knowledge by answering questionnaires based on the section's material.

#### 3.1.4 Learning objectives

##### Objective 1 – Interpreting community ecology plots

In section one, students are guided to produce boxplots that summarize the abundance of tree species that were impacted by the outbreak (Figure 2a). In section 2, students are asked to make bar plots visualizing the health status of the various tree species during the outbreak (Figure 2a). These exercises in data visualization reveal which tree species were impacted most by the hemlock looper, thus describing the tree community after the outbreak.

##### Objective 2 – Assess specialist vs. generalist species status with data visualization

In section three, students are encouraged to develop hypotheses and predictions about the hemlock looper's tree preferences and use the data to draw conclusions as to whether the hemlock looper is a specialist or generalist species (i.e., whether it feeds on all trees equally or prefers one or two species) based on the plots they produced in the previous sections. Students are then introduced to a graphical method to test their hypotheses, which consists of a scatterplot with a 1:1 line displaying the relative abundance of trees in each species (*x*) and the proportion of trees of each species that are dead (*y*; Figure 2b). The position of the points along the 1:1 line is used to determine whether the hemlock looper has preferences for certain species and conclude whether it is a specialist insect. Students are challenged to infer that points falling above the 1:1 line indicate that the hemlock looper attacks proportionately more of these trees, even if their relative abundance is lower than other trees.

##### Objective 3 – Data interpretation for data‐driven management recommendations

In section four, students are encouraged to make data‐driven management recommendations using inferences drawn from the data (Figure 2c). This section includes three videos focusing on a contemporary hemlock looper outbreak in Metro Vancouver, with a few guiding questions for students to reflect upon while they watch the clips. To provide flexibility for discussions on the socio‐ecological ramifications of a hemlock looper outbreak, the tutorial asks students to reply to a prompt, which is to be submitted to their instructor. The tutorial closes with examples of big‐picture ideas on the biological, social, and ethical factors to consider in management and provides some main takeaways and additional references for students to pursue at their leisure.

#### 3.1.5 Potential tutorial applications

This tutorial was designed for use in a first‐ or second‐year ecology course/curriculum that has previously discussed species interactions, in particular consumer‐resource interactions, and aims to provide students with a real‐life, local example of the impact pests can have on ecological communities. The tutorial can also be used to expose students to data‐driven hypothesis testing, as we use the dataset to ask the question “does the hemlock looper have a preferred tree prey species?” allowing students to apply the scientific method in a more computationally‐intensive environment as opposed to a lab environment. This tutorial provides students with the opportunity to develop their analytical skills as they produce plots and interpret data and then apply these skills to make data‐driven management recommendations. Overall, this tutorial aims to encourage undergraduate students to see consumer‐resource dynamics from a more holistic lens, both from an ecological and a socio‐ecological perspective, while introducing students to important open science practices such as open data and technical skills using an open‐source programming language (*R*).

### 3.2 Tutorial example 2. Understanding biodiversity with metrics & rank abundance curves

#### 3.2.1 Tutorial overview

In this tutorial, we present an introduction to biodiversity indices and biodiversity change using a dataset of benthic stream invertebrates collected in the Turkey Lakes Watershed in Ontario, Canada. We use a publicly available dataset that contains measurements of benthic invertebrate abundance and diversity in streams near areas that have been historically impacted by logging (Government of Canada, 2020). This tutorial aims to expose students to core biodiversity concepts (see tutorial section one: “Why biodiversity matters”), common ways to collect biodiversity data (see tutorial section two: “Types of data typically collected”), methods of quantifying biodiversity change (see tutorial section three: “Real world biodiversity data” and section four: “Metrics of biodiversity”), and ways to compare biodiversity change across time and environmental conditions (see tutorial section five: “Visualizing biodiversity”). In addition, this tutorial teaches students how to calculate biodiversity indices via the “codyn” *R* package (containing pre‐packaged functions to quantify biodiversity change; Hallett et al., 2020) and provides an activity for students to calculate biodiversity indices themselves in different logging areas (see tutorial section six: “Measure and visualize biodiversity with codyn”). This tutorial thus presents ways to quantify and interpet how anthropogenic activities influence biodiversity change in the real world.

#### 3.2.2 Data collection background

In 1997, an experiment was conducted in the Turkey Lakes watershed where sites were logged with varying intensities (low, medium, and high) to test how logging affected stream ecosystems. From 1995 to 2009, scientists collected, identified, and counted benthic invertebrates from streams near these logged areas around the Turkey Lakes watershed. Benthic invertebrates are typically small, aquatic animals and the aquatic larval stages of insects that form an important link between aquatic and terrestrial habitats and can act as an indicator of ecosystem health. The data are available on the Government of Canada's open data portal (Government of Canada, 2020).

#### 3.2.3 Description of the activity

Using the benthic stream invertebrates from the Turkey Lakes Watershed as a case study, the tutorial covers ecological concepts and skills such as calculating species richness, evenness, and other biodiversity metrics, and visualizing rank abundance curves. The tutorial consists of six sections that address three distinct learning objectives (featured below). Using the “show code” button, students are able to follow along with well‐annotated *R* code that explores the data, creates plots, and calculates biodiversity indices. At the end of the tutorial, students can complete an additional self‐guided activity that applies these coding skills to analyze the Turkey Lakes Watershed dataset.

#### 3.2.4 Learning objectives

##### Objective 1 – Identify, calculate, and implement commonly used metrics to quantify biodiversity

In section one, students are given an introduction to why biodiversity change is important for conservation decisions and resource management, and the potential ecological implications of biodiversity change for disrupting ecosystem dynamics. In sections two and three, students are introduced to the raw data typically collected to quantify biodiversity and an example of real‐world biodiversity data with the Turkey Lakes Watershed data. In section four, students are then presented with a suite of indices to quantify biodiversity change (e.g., species richness, Shannon‐Wiener Index (H′), Simpson's Diversity Index (D), evenness (E)), using the Turkey Lakes Watershed data to quantify these indices (Figure 3a). These exercises in estimating biodiversity change reveal annual changes in community composition and allow students to begin to evaluate the role of logging on those changes.

##### Objective 2 – Understand and create figures used to display biodiversity data

In section five, students are presented with ways to visualize changes in biodiversity using rank‐abundance curves (RAC), using select data from the Turkey Lakes Watershed (Figure 3b). Students are then able to compare RACs to trace species distributions across study sites and logging intensity treatments. Visualizing the different ways RACs can be used to inform changes in ecosystem dynamics gives students exposure to the tools that could be used to inform management and conservation decisions.

##### Objective 3 – Analyze biodiversity change in benthic invertebrates over multiple years and disturbance levels

In section 6, students are introduced to the “codyn” R package (Hallett et al., 2020), which includes built‐in functions to quantify changes in biodiversity after logging and during forest recovery by calculating a suite of biodiversity indices for stream invertebrates over time and between logging treatments. At the end of the tutorial, students have the opportunity to apply these skills with an activity exploring in‐depth questions about the impact of logging intensity on invertebrate biodiversity in the Turkey Lakes Watershed (Figure 3c).

#### 3.2.5 Potential tutorial applications

This tutorial was designed for use in a third‐ or fourth‐year ecology course that has previously covered aspects of community ecology, in particular biodiversity indices, and aims to provide students with a real‐life example of how biodiversity can change over time and with anthropogenic activities. The tutorial exposes students to how discipline‐specific *R* packages like “codyn” can be used to analyze publicly available datasets like the Turkey Lakes Watershed stream dataset. This tutorial teaches foundational biodiversity metrics used in community ecology, applies these indices to quantify important changes in community dynamics, and considers potential implications for management when it comes to logging impacts on biodiversity. Overall, this tutorial uses well‐documented R Markdown files and a Shiny App to teach important skills in biodiversity science and encourages application of these skills by working with open data and open source programming languages like *R*.

## 4 DISCUSSION

We designed open educational resources in the form of online tutorials to support teaching undergraduates a variety of concepts in ecology and evolutionary biology (EEB) while integrating open science skills. The tutorials are portable and ready to use, allowing instructors to integrate them into their classroom learning objectives. The tutorials provide hands‐on experience in data manipulation and statistical analysis and introduce students to real‐world open data and open‐source software. Here, we highlighted two tutorials to provide examples of how such data can be used to teach EEB concepts and can be applied to management decisions. Our aim with these tutorials was to increase students' enthusiasm and confidence in using and interpreting publicly available data while learning fundamental EEB concepts. The tutorials cater to a variety of student proficiency levels and provide a scaffolding for instructors to incorporate programming into their own coursework.

To reduce cognitive load, the tutorials begin by presenting relevant information on the biological concepts that are covered and clearly present tutorial learning objectives. We designed the tutorials in a way that breaks information down into smaller “chunks” to improve the absorption and retention of information, deep comprehension, and student application of knowledge (Kossen & Ooi, 2021). For example, the tutorials that we highlighted in this article are formatted with different tabs or headings that allow students to work through them in shorter sections. In addition, the tutorials are scaffolded so students can learn a biological concept or data science skill and then apply this new information by answering questions or programming directly in an R Markdown file. This scaffolding approach to teaching can help reduce the learner's cognitive load and allows them to perform tasks that they may not have been able to otherwise (Myhill & Warren, 2005; Turner & Berkowitz, 2005; Van Merrienboer et al., 2003). We designed the tutorials to move from simple to more complex tasks, used design features to draw attention to key elements (e.g., bolded text), and incorporated visuals and interactive plots to keep learners engaged. Moreover, we used a flexible tutorial format to allow students to engage with the programming elements to different degrees (e.g., view or hide code segments) (Clark et al., 2006; Van Merrienboer et al., 2003).

We developed these tutorials to cater to different programming and statistical proficiency levels that may be encountered among students in undergraduate EEB programs across the world. Since students in EEB programs may have minimal background in programming and statistics, there can be a trade‐off between time spent learning to code versus learning ecological concepts (Auker & Barthelmess, 2020). To address this, the tutorials vary in exposure to and interaction with programming, from no coding requirements in the case of a Shiny App, to options to view code when visualizing data or conducting statistical analyses, to downloading an R Markdown file and following prompts to practice coding by manipulating the code. The opportunity to engage with and learn programming at different levels varies both between and within the tutorials presented in this article. Furthermore, our tutorials use a range of statistical concepts, from simple summary statistics to more complex statistical modeling. Self‐directed tutorials combining data science practices (i.e., data visualization and statistics) with concrete questions and working examples have been shown to help students integrate ecological and statistical concepts alongside programming skills (O'Reilly et al., 2017; Styers et al., 2021). By accommodating a variety of proficiency levels and conceptual backgrounds, we provide an entry point for a wide range of undergraduate students to learn foundational concepts in EEB and open science.

There may be additional challenges in implementing our tutorials in a range of contexts around the world as multiple barriers (e.g., language, computing, financial, social, etc.) have been shown to exacerbate inequalities in the global accessibility of open science (Bezuidenhout et al., 2017; Sandersan, 2020; Staunton et al., 2021). For instance, because the data in our tutorials are set in Canada, the additional benefits of working with local data will not apply to all audiences (Serwadda et al., 2018). In addition, the ability to use local open data for teaching varies geographically, as higher income countries tend to publish more open access data (Serwadda et al., 2018), and our teaching material may thus appeal more to students from the global North. Yet, the topics covered in most of our tutorials (e.g., biodiversity, community ecology, sexual selection) should appeal to a broad audience. Ultimately, we hope that with the scaffolding provided in our tutorials, the format and content will be easily accessible and adaptable to different data and contexts that may be more familiar to different regions of the world.

Skills such as the ability to manipulate and analyze large datasets with open‐source software are becoming increasingly important within and outside of academia (Lai et al., 2019; Michener & Jones, 2012). However, designing assignments involving programming requires a significant time investment, such that incorporating programming in undergraduate curricula can be challenging for instructors (Auker & Barthelmess, 2020; Morrison et al., 2020). Preparing undergraduate students for research, including discussions about open science, can require additional planning and in‐class time (Morrison et al., 2020). The tutorials presented here may help reduce this time investment for instructors by both providing user‐friendly resources to directly incorporate into their curriculum and scaffolding on which to expand or develop additional tutorials that are shaped to the needs of individual courses. Thus, instructors without a significant background in data science could easily implement our tutorials in their classrooms. Moreover, our additional set of resources (e.g., guiding questions, sample lesson plan, etc.) on the tutorial website is designed to assist instructors with integrating these tutorials into their courses (see “Guidelines for instructors” tab on the website).

In conclusion, the tutorials presented here teach key EEB concepts via publicly available datasets and statistical analyses using *R* software in a pre‐packaged interactive way. This approach may help reduce the cognitive load for students and the time needed for instructors to create course content. In doing so, these tutorials help to bridge the gap between open science research and open science teaching practices by using publicly available datasets and open‐source statistical software.

## AUTHOR CONTRIBUTIONS

**Jory E. Griffith:** Software (equal); writing – original draft (lead); writing – review and editing (lead). **Elizabeth Houghton:** Software (equal); visualization (lead); writing – original draft (lead); writing – review and editing (lead). **Margaret A. Slein:** Software (equal); visualization (lead); writing – original draft (lead); writing – review and editing (lead). **Maxime Fraser Franco:** Software (equal); writing – original draft (lead); writing – review and editing (lead). **Jhoan Chávez:** Software (equal); writing – review and editing (supporting). **Amy B. Forsythe:** Software (equal); writing – review and editing (supporting). **Victoria M. Glynn:** Software (equal); writing – original draft (supporting); writing – review and editing (supporting). **Egor Katkov:** Software (equal); writing – review and editing (supporting). **Kirsten M. Palmier:** Software (equal); writing – original draft (supporting); writing – review and editing (supporting). **Zihaohan Sang:** Software (equal); writing – review and editing (supporting). **Rolando Trejo‐Pérez:** Software (equal); writing – review and editing (supporting). **Bryn Wiley:** Software (equal); writing – review and editing (supporting). **Jennifer M. Sunday:** Conceptualization (lead); funding acquisition (equal); project administration (lead); resources (equal); supervision (lead); writing – review and editing (equal). **Joey R. Bernhardt:** Conceptualization (lead); funding acquisition (equal); project administration (lead); resources (equal); supervision (lead); writing – review and editing (equal).

## FUNDING INFORMATION

The Living Data Project is funded by a Collaborative Research and Training Experience (CREATE) from the Natural Sciences and Engineering Research Council of Canada (NSERC) and the Canadian Institute of Ecology and Evolution (CIEE).

## CONFLICT OF INTEREST STATEMENT

The authors declare that there are no competing interests.

## Data Availability

Data used in the tutorial presented in this article are available as follows: Tree Swallow Life History: R Basics & Data Exploration and Sexual Dimorphism & Sexual Selection in Tree Swallows tutorial – https://doi.org/10.6084/m9.figshare.14156801.v1; Community Ecology: Hemlock Looper Outbreak on Anticosti Island tutorial – Government of Canada. Record ID: 9dda09b0‐649f‐4002‐b207‐7b204eb81cbb, https://open.canada.ca/data/en/dataset/9dda09b0‐649f‐4002‐b207‐7b204eb81cbb; Tree Diversity & Dendrochronology in Canadian Boreal Forests tutorial – Borealis. https://doi.org/10.5683/SP3/PZCAVE; Disease Modelling in Mule Deer tutorial – Government of Alberta: https://www.alberta.ca/chronic‐wasting‐disease‐history‐in‐alberta.aspx#jumplinks‐0; Understanding Biodiversity with Metrics & Rank Abundance Curves – Government of Canada. Record ID: f2ac0ae9‐dd2f‐4a70‐b059‐f8a49d9f5982, https://open.canada.ca/data/en/dataset/f2ac0ae9‐dd2f‐4a70‐b059‐f8a49d9f5982.
